# Gut microbiome metagenomic sequences of honey bees (*Apis mellifera*) exposed to crops

**DOI:** 10.1128/mra.00731-24

**Published:** 2025-02-04

**Authors:** L. Tran, L. Lansing, M. Cunningham, J. Ho, T. Deckers, T. Newman, L. Wu, A. S. Gregoris, J. Zorz, L. Muntz, K. Lee, D. Trépanier-Leroux, I. M. Conflitti, M. Pepinelli, E. M. Walsh, N. Morfin, J. E. Powell, N. Moran, S. E. Hoover, S. F. Pernal, R. W. Currie, P. Giovenazzo, E. Guzman-Novoa, H. Jabbari, L. J. Foster, A. Zayed, R. Ortega Polo, M. M. Guarna

**Affiliations:** 1 Agriculture and Agri-Food Canada, Beaverlodge Research Farm, Beaverlodge, Alberta, Canada; 2 Agriculture and Agri-Food Canada, Lethbridge Research and Development Centre, Lethbridge, Alberta, Canada; 3 Department of Computer Science, University of Victoria 8205 , Victoria, British Columbia, Canada; 4 Department of Biology, York University 7991 , Toronto, Ontario, Canada; 5 School of Environmental Sciences, University of Guelph 3653 , Guelph, Ontario, Canada; 6 Department of Integrative Biology, University of Texas, Austin, Texas, USA; 7 Department of Biological Sciences, University of Lethbridge, Lethbridge, Alberta, Canada; 8 Department of Entomology, University of Manitoba 8664 , Winnipeg, Manitoba, Canada; 9 Département de biologie, Université Laval, Québec City, Québec, Canada; 10 Department of Biochemistry and Molecular Biology, University of British Columbia, Vancouver, British Columbia, Canada; Montana State University, Bozeman, Montana, USA

**Keywords:** honey bees, metagenomics, gut microbiome

## Abstract

The gut microbiome of the European honey bee (*Apis mellifera*) is vital to its health, yet large-scale studies are scarce. We present metagenomic sequencing data from 180 samples collected near and far from eight crops across Canada over 2 years. These data sets will help address various biological and environmental questions.

## ANNOUNCEMENT

Microbiomes play a crucial role in agroecosystems as well as in individual animal and plant health (1, 2). European honey bees (*Apis mellifera*) rely heavily on their gut microbiome for nutrition, health, and immunity (3). However, large-scale studies are still scarce. We announce the release of shotgun metagenomic sequencing data from 180 honey bee samples collected near and far from eight different crops (apple, canola oil, canola seed, corn, cranberry, highbush blueberry, lowbush blueberry, and soybean) across Canada in 2020 and 2021 (Table 1). Four honey bee colonies were placed at five sites near to, or far from, each crop as previously described (4, 5). We generally defined “near” as within 0.5 km of the crop and “far” as greater than 1.5 km from the crop.

**TABLE 1:** Agricultural crops, sampling year, and province – region from which the experimental bee colonies were sampled (4, 5)^*a*^

Crop	Years	Crop sample prefix	Province – region
Apple	2021	APP	Québec – Montérégie
Corn	2020	COR	Southwestern Ontario
Cranberry	2020, 2021	CRA	British Columbia – Fraser Valley, Québec – Centre-du-Québec
Canola oil	2020, 2021	CAC	Northern Alberta Southern Alberta Southern Manitoba
Lowbush blueberry	2021	LBB	Québec Saguenay – Lac-Saint-Jean Region
Soybean	2020	SOY	Southern Manitoba
Canola seed	2020, 2021	CAS	Southern Alberta
Highbush blueberry	2020, 2021	HBB	British Columbia – Fraser Valley

^*a*^ Our metadata includes GPS locations and sample names encoded with experimental details; a three-letter code specifying the agricultural crop (as per the ‘crop sample prefix’ column) followed by a two-digit site number (from 01 to 05 for 2020 and from 06 to 10 for 2021) and ending with an “e” or “u” indicating whether the sampled bees were near (e = exposed) or far (u = unexposed) from the named crop. In the case of NCBI BioProject PRJNA1124990, the time point (T1–T4) is also indicated as part of the sample name.

For highbush blueberry, bees were collected at four time points: before (time point 1), during (time point 2), at the end of (time point 3), and after (time point 4) the pollination period (4). For the other crops, bees for microbiome analyses were collected only at time point 2. Bee samples were processed by isolating whole guts, i.e., digestive tracts excluding the honey crop. Each replicate consisted of pooled guts from 12 individual bees (i*.*e., three bees from each of the four colonies located at each site). After homogenization, samples were shipped on dry ice to the Centre d’expertise et des services Génome Québec/Génome Québec Centre of Expertise and Services (Montréal, Quebec, Canada) for genomic DNA extraction and quantification (Quant-iT PicoGreen dsDNA Assay Kit, Life Technologies), library construction (NEBNext Ultra II DNA Library Prep Kit for Illumina, New England Biolabs) including library size selection (SparQ beads, Qiagen) and quantification (Kapa Illumina GA with Revised Primers-SYBR Fast Universal Kit, Kapa Biosystems), and average fragment size determination (LabChip GX Touch Nucleic Acid Analyzer, PerkinElmer). Sequencing was performed on an Illumina NovaSeq 6000, generating 151 base pair paired-end reads. The samples yielded raw sequencing reads ranging from 38,474,441 to 133,152,604 reads.

For taxonomic classification, sequences were trimmed of adapters and subjected to quality control using *fastp* (v0.23.2) (6), aligned to the PhiX bacteriophage (GenBank accession NC_001422.1) using *Bowtie2* (v2.5.1) (7) with argument --un-conc-gz to retain non-PhiX sequences, then classified against bacterial genomes within the curated BeeRoLaMa database (8) using Kraken 2 (v2.1.2) (9) with argument --paired. Default parameters were used except where otherwise noted. Classified counts confirmed the expected core honey bee gut microbiome taxa as well as the occurrence of pathogens *Paenibacillus* spp., *Spiroplasma* spp., and *Melissococcus plutonius*.

Our data sets should prove useful for answering diverse biological and environmental questions regarding differences in microbial diversity and relative abundance across Canada over 2 years. Some of the pathogen, chemical, and pollen diversity data associated with these colonies have been reported (4, 5, 10–12) and can be used to study interactions between these factors and the bee gut microbiome composition. The availability of the metagenomic data sets announced here will also facilitate association analyses between microbiome composition and environmental conditions, and inform the use of honey bees and their microbiomes for environmental monitoring (2).

## Data Availability

The raw metagenome sequences for all T2 samples are available as BioProject PRJNA999720 in the NCBI BioProject database (https://www.ncbi.nlm.nih.gov/bioproject), and sequences for all time points for the HBB experiment are available as NCBI BioProject PRJNA1124990. Both BioProjects fall under the umbrella BioProject PRJNA1033630.

## References

[1] Liu X (Christine), Floate KD, Gorzelak MA, Holman DB, Hrycauk S, Kubota H, Lupwayi N, Neilson JAD, Ortega Polo R, Petri RM, Tran L, Wang H, Wilches D, Yang X, Zorz J, Guarna MM. 2023. Prairie agroecosystems: interconnected microbiomes of livestock, soil and insects. Agr 13:326. doi:10.3390/agriculture13020326

[2] Cunningham MM, Tran L, McKee CG, Ortega Polo R, Newman T, Lansing L, Griffiths JS, Bilodeau GJ, Rott M, Guarna MM. 2022. Honey bees as biomonitors of environmental contaminants, pathogens, and climate change. Ecol Indic 134:108457. doi:10.1016/j.ecolind.2021.108457

[3] Motta EVS, Moran NA. 2024. The honeybee microbiota and its impact on health and disease. Nat Rev Microbiol 22:122–137. doi:10.1038/s41579-023-00990-3 38049554 PMC10998682

[4] McAfee A, French SK, Wizenberg SB, Newburn LR, Tsvetkov N, Higo H, Common J, Pernal SF, Giovenazzo P, Hoover SE, Guzman-Novoa E, Currie RW, Veiga PW, Conflitti IM, Pepinelli M, Tran L, Zayed A, Guarna MM, Foster LJ. 2024. Higher prevalence of sacbrood virus in Apis mellifera (Hymenoptera: Apidae) colonies after pollinating highbush blueberries. J Econ Entomol 117:1324–1335. doi:10.1093/jee/toae119 38877967 PMC11318621

[5] McAfee A, Alavi-Shoushtari N, Tran L, Labuschagne R, Cunningham M, Tsvetkov N, Common J, Higo H, Pernal SF, Giovenazzo P, Hoover SE, Guzman-Novoa E, Currie RW, Veiga PW, French SK, Conflitti IM, Pepinelli M, Borges D, Walsh EM, Bishop CA, Zayed A, Duffe J, Foster LJ, Guarna MM. 2024. Climatic predictors of prominent honey bee (Apis mellifera) disease agents: Varroa destructor, Melissococcus plutonius, and Vairimorpha spp. PLOS Clim 3:e0000485. doi:10.1371/journal.pclm.0000485

[6] Chen S. 2023. Ultrafast one-pass FASTQ data preprocessing, quality control, and deduplication using fastp. Imeta 2:e107. doi:10.1002/imt2.107 38868435 PMC10989850

[7] Langmead B, Salzberg SL. 2012. Fast gapped-read alignment with Bowtie 2. Nat Methods 9:357–359. doi:10.1038/nmeth.1923 22388286 PMC3322381

[8] Ortega Polo R, Tran L, Guarna M. 2023. BeeRoLaMa v1 honey bee microbiota database (1.0). Zenodo. 10.5281/zenodo.8043866.

[9] Wood DE, Lu J, Langmead B. 2019. Improved metagenomic analysis with Kraken 2. Genome Biol 20:257. doi:10.1186/s13059-019-1891-0 31779668 PMC6883579

[10] French SK, Pepinelli M, Conflitti IM, Jamieson A, Higo H, Common J, Walsh EM, Bixby M, Guarna MM, Pernal SF, Hoover SE, Currie RW, Giovenazzo P, Guzman-Novoa E, Borges D, Foster LJ, Zayed A. 2024. Honey bee stressor networks are complex and dependent on crop and region. Curr Biol 34:1893–1903. doi:10.1016/j.cub.2024.03.039 38636513

[11] Bixby M, French SK, Wizenberg SB, Jamieson A, Pepinelli M, Cunningham MM, Conflitti IM, Foster LJ, Zayed A, Guarna MM. 2024. Identifying and modeling the impact of neonicotinoid exposure on honey bee colony profit. J Econ Entomol 117:2228–2241. doi:10.1093/jee/toae227 39436769 PMC11682944

[12] Wizenberg SB, Newburn LR, Richardson RT, Pepinelli M, Conflitti IM, Moubony M, Borges D, Guarna MM, Guzman-Novoa E, Foster LJ, Zayed A. 2023. Environmental metagenetics unveil novel plant-pollinator interactions. Ecol Evol 13:e10645. doi:10.1002/ece3.10645 37941738 PMC10630067

